# Supplementary figures and images for: Proteasome-Mediated Regulation of Cdhr1a by Siah1 Modulates Photoreceptor Development and Survival in Zebrafish
Source: Front Cell Dev Biol. 2020 Nov 23;8:594290. doi: 10.3389/fcell.2020.594290 (PMC7719784; doi:10.3389/fcell.2020.594290)

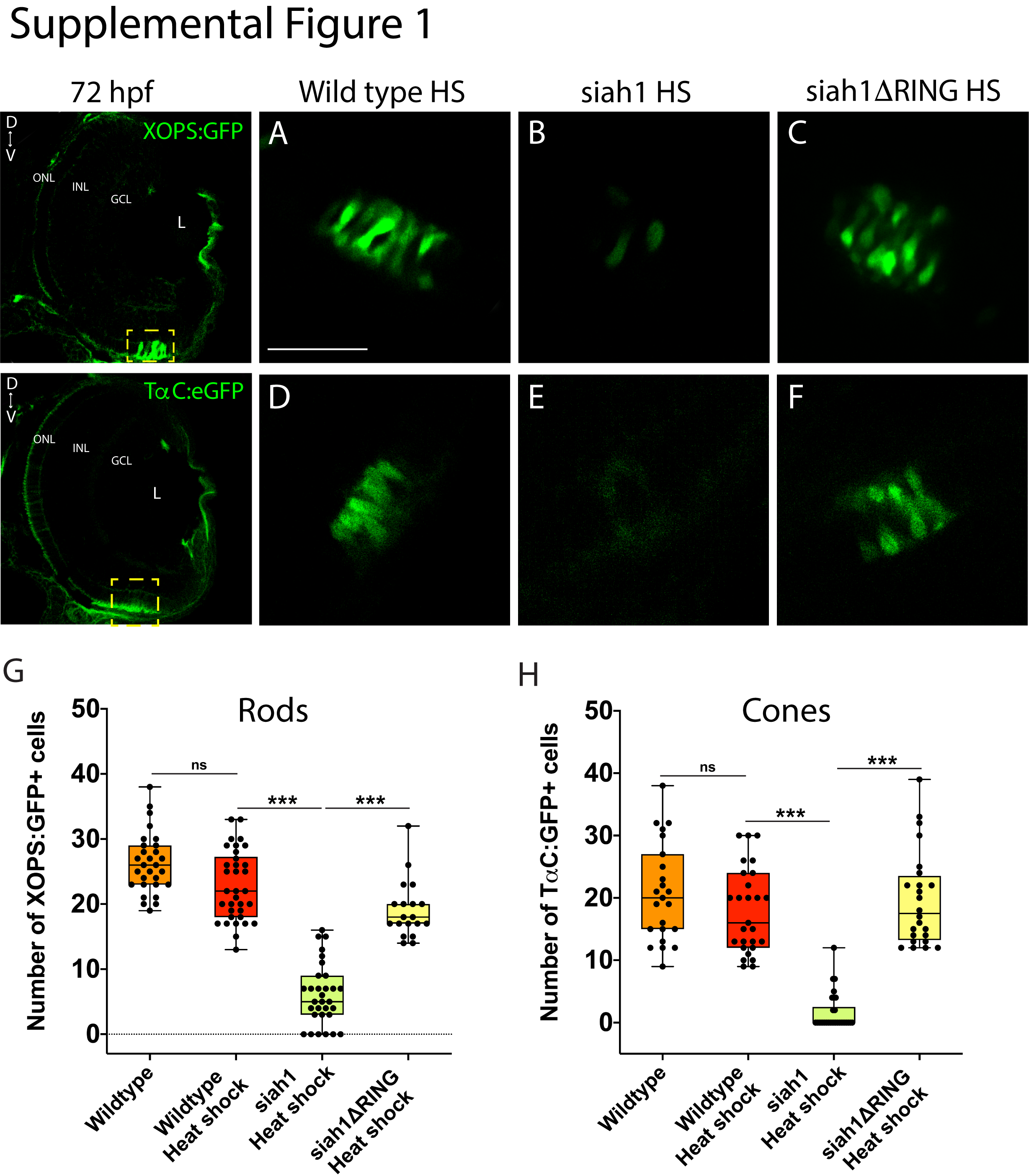

Supplement: Supplementary Figure 1 — Siah1 overexpression decreases the number of rods and cones. Confocal stacks of heat shocked (HS) Tg[XOPS:GFP] (wildtype), Tg[hsp70:siah1]/Tg[XOPS:GFP] (siah1), and Tg[hsp70:siah1ΔRING]/Tg[XOPS:GFP], (siah1ΔRING) embryos were collected analyzed in 3D for GFP fluorescence (A–C). Region analyzed and presented is outlined in yellow. Overexpression of Siah1 resulted in significantly fewer GFP + rod cells (G). Confocal stacks of heat shocked (HS) Tg[TαC:eGFP] (wildtype), Tg[hsp70:siah1]/Tg[TαC:eGFP] (siah1), and Tg[hsp70:siah1ΔRING]/Tg[TαC:eGFP], (siah1ΔRING) embryos were analyzed in 3D for GFP fluorescence (D–F). Region analyzed and presented is outlined in yellow. Overexpression of Siah1 resulted in significantly fewer GFP + cone cells (H). Scale bar = 50 μm. [file Image_1.TIF]

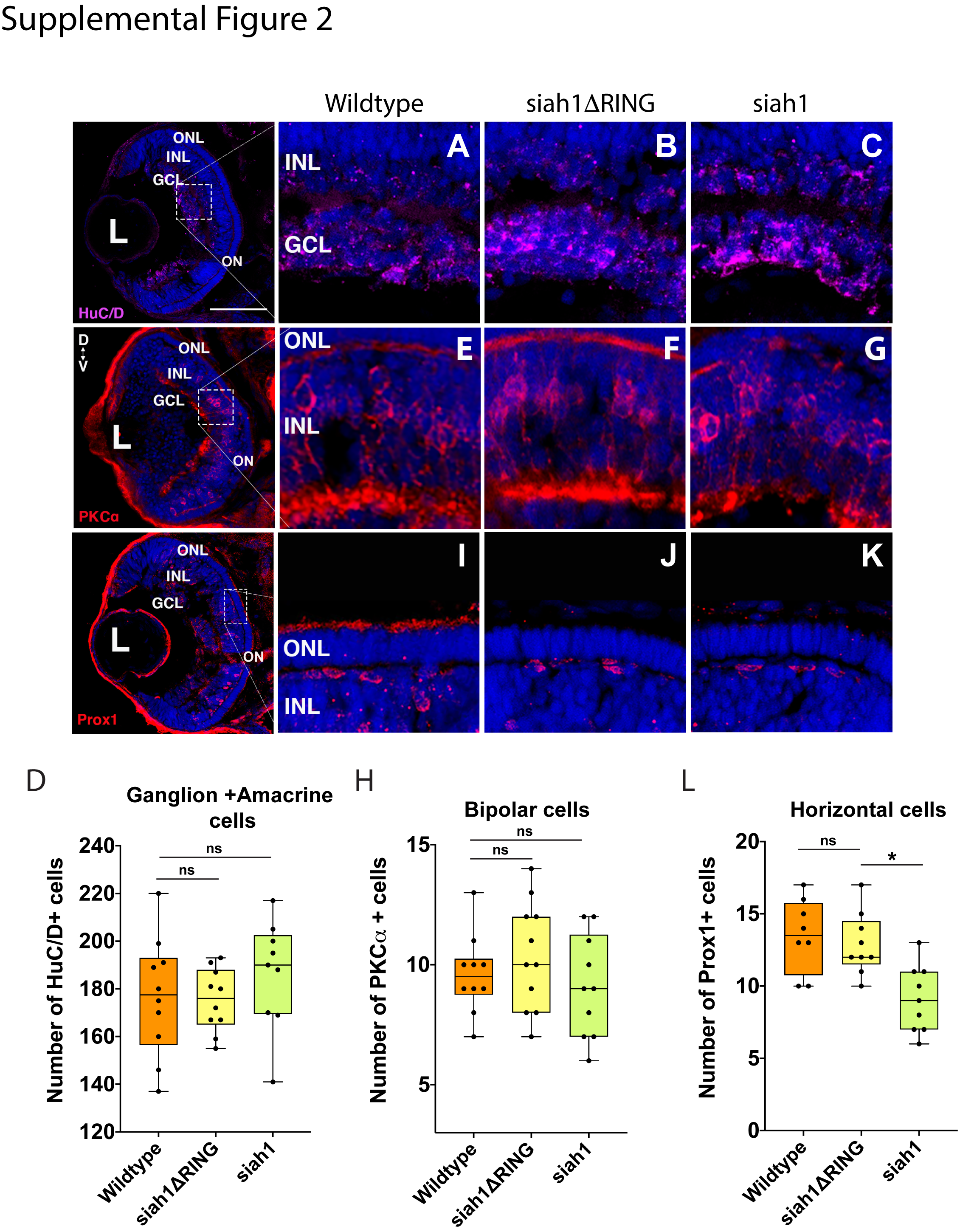

Supplement: Supplementary Figure 2 — Inner retinal neurons are not affected by Siah1 overexpression. Retinal cryosections from wildtype, Tg[hsp70:siah1] (siah1), and Tg[hsp70:siah1ΔRING] (siah1ΔRING) embryos heat shocked (HS) and analyzed for effects on retinal inner neurons using IHC. Retinal ganglion and amacrine cells were visualized and quantified using Huc/D staining (A–D). Bipolar cells were visualized and quantified using PKCα (E–H). Horizontal cells were observed and quantified using prox1 staining (I–L). DNA was stained with DAPI (blue). Scale bar = 50 μm. L, lens; ONL, outer nuclear layer; INL, Inner nuclear layer; GCL, ganglion cell layer; ON, optic nerve; D, Dorsal; and V, Ventral. [file Image_2.TIF]

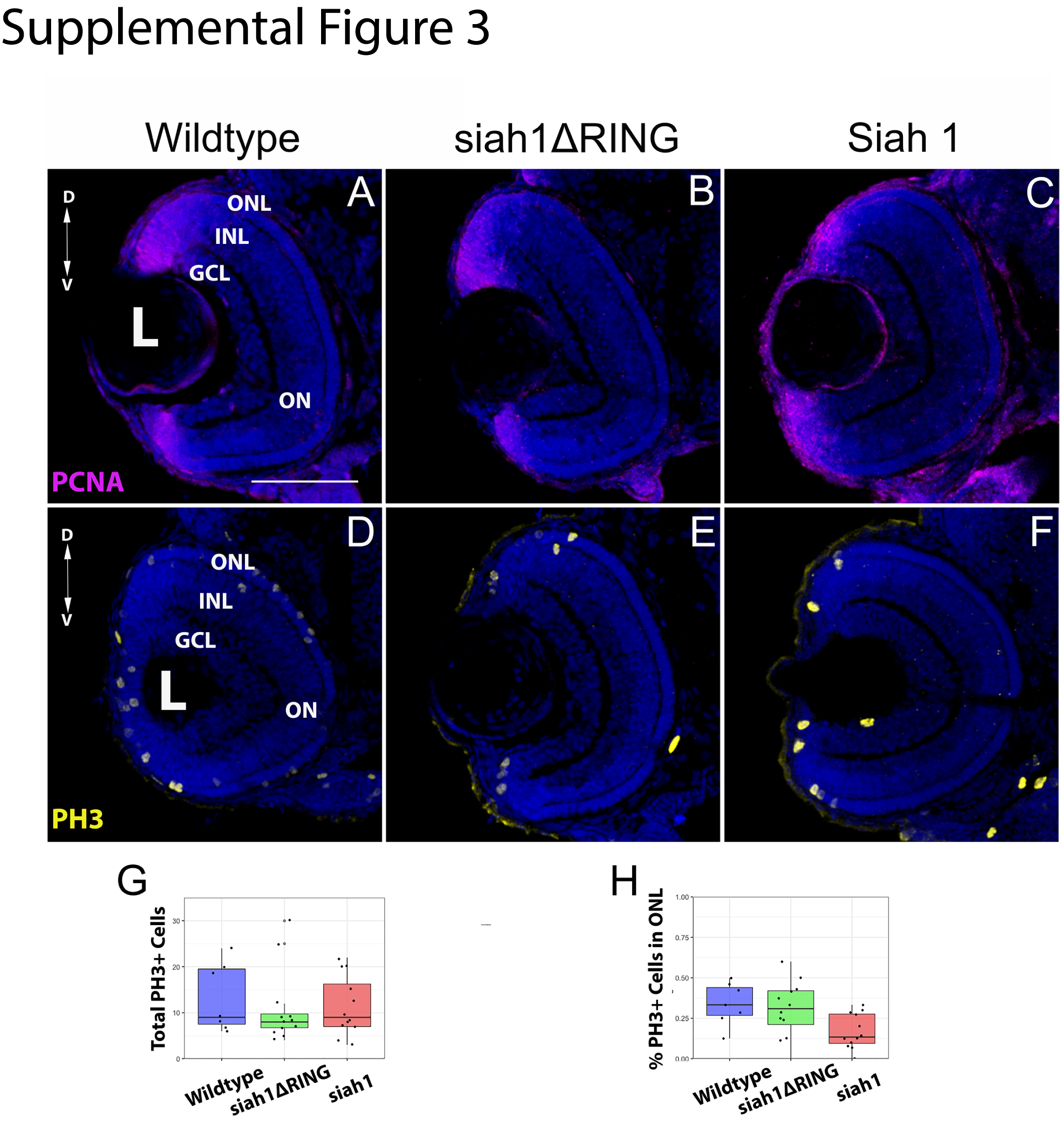

Supplement: Supplementary Figure 3 — Siah1 does not affect retinal cell proliferation or cell cycle progression. Retinal cryosections from wildtype, Tg[hsp70:siah1] (siah1), and Tg[hsp70:siah1ΔRING] (siah1ΔRING) embryos heat shocked (HS) and analyzed for cell cycle status using PCNA (A–C) and PH3 (D–F) IHC staining. Number of PCNA or PH3 positive cells did not significantly change upon Siah1 overexpression (G,H). DNA was stained with DAPI (blue). Scale bar = 50 μm. L, lens; ONL, outer nuclear layer; INL, Inner nuclear layer; GCL, ganglion cell layer; ON, optic nerve; D, Dorsal; and V, Ventral. [file Image_3.TIF]

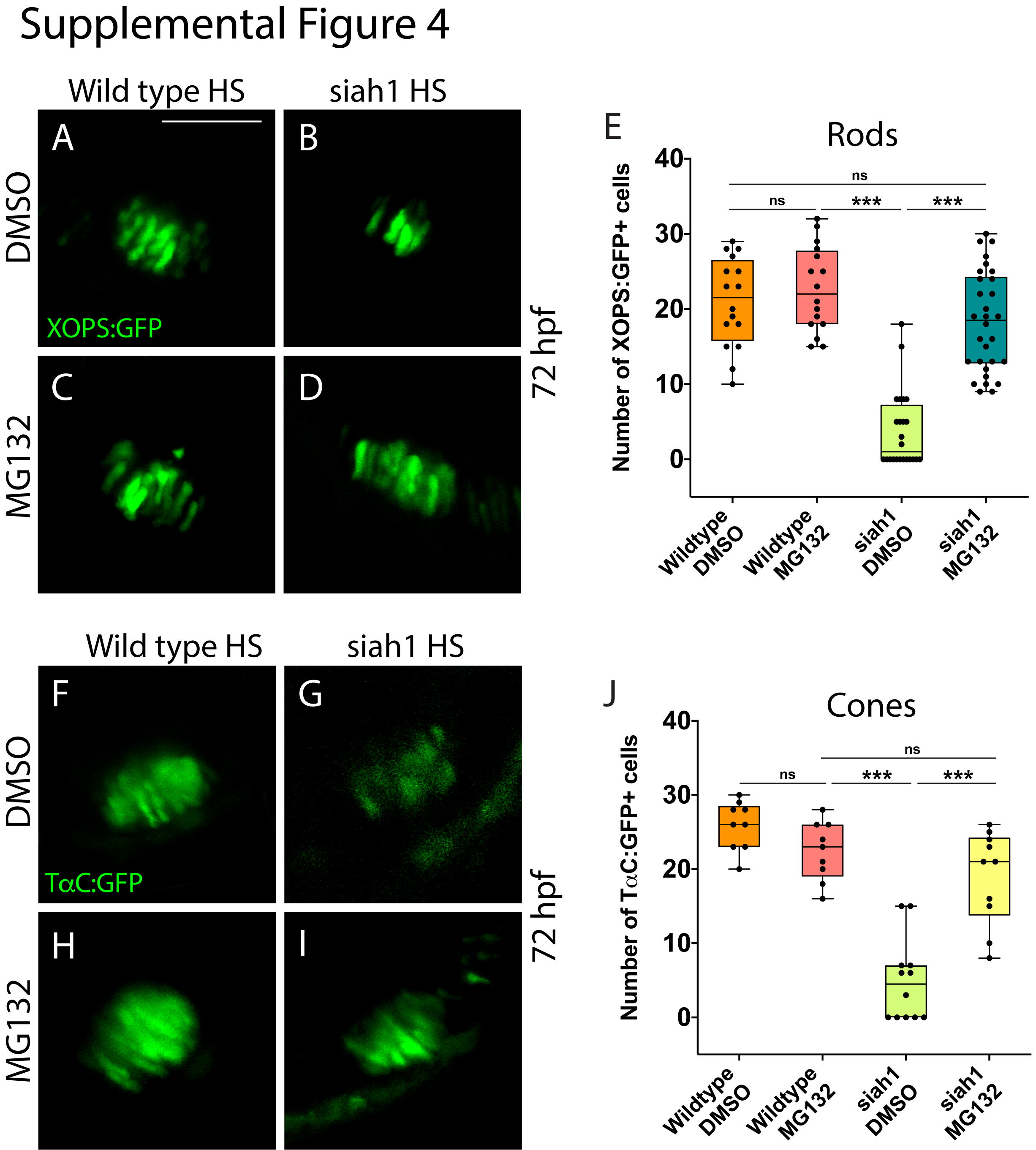

Supplement: Supplementary Figure 4 — Proteasome inhibition rescues Siah1 overexpression phenotype. Confocal stacks of heat shocked (HS) Tg[XOPS:GFP] (wildtype), Tg[hsp70:siah1]/Tg[XOPS:GFP] (siah1), and Tg[hsp70:siah1ΔRING]/Tg[XOPS: GFP], (siah1ΔRING) embryos treated with DMSO or MG132 were collected analyzed in 3D for GFP fluorescence. Quantification was restricted to the ventral retina (A–D). Treatment with MG132 prevented a significant decrease in GFP + rod cells compared to DMSO in siah1 HS embryos (E). Confocal stacks of heat shocked (HS) Tg[TαC:eGFP] (wildtype), Tg[hsp70:siah1]/Tg[TαC:eGFP] (siah1), and Tg[hsp70:siah1ΔRING]/Tg[TαC:eGFP], (siah1ΔRING) embryos treated with DMSO or MG132 were analyzed in 3D for GFP fluorescence. Quantification was restricted to the ventral retina (F–I). Treatment with MG132 prevented a significant decrease in GFP + cone cells compared to DMSO in siah1 HS embryos (J). Scale bar = 50 μm. [file Image_4.TIF]

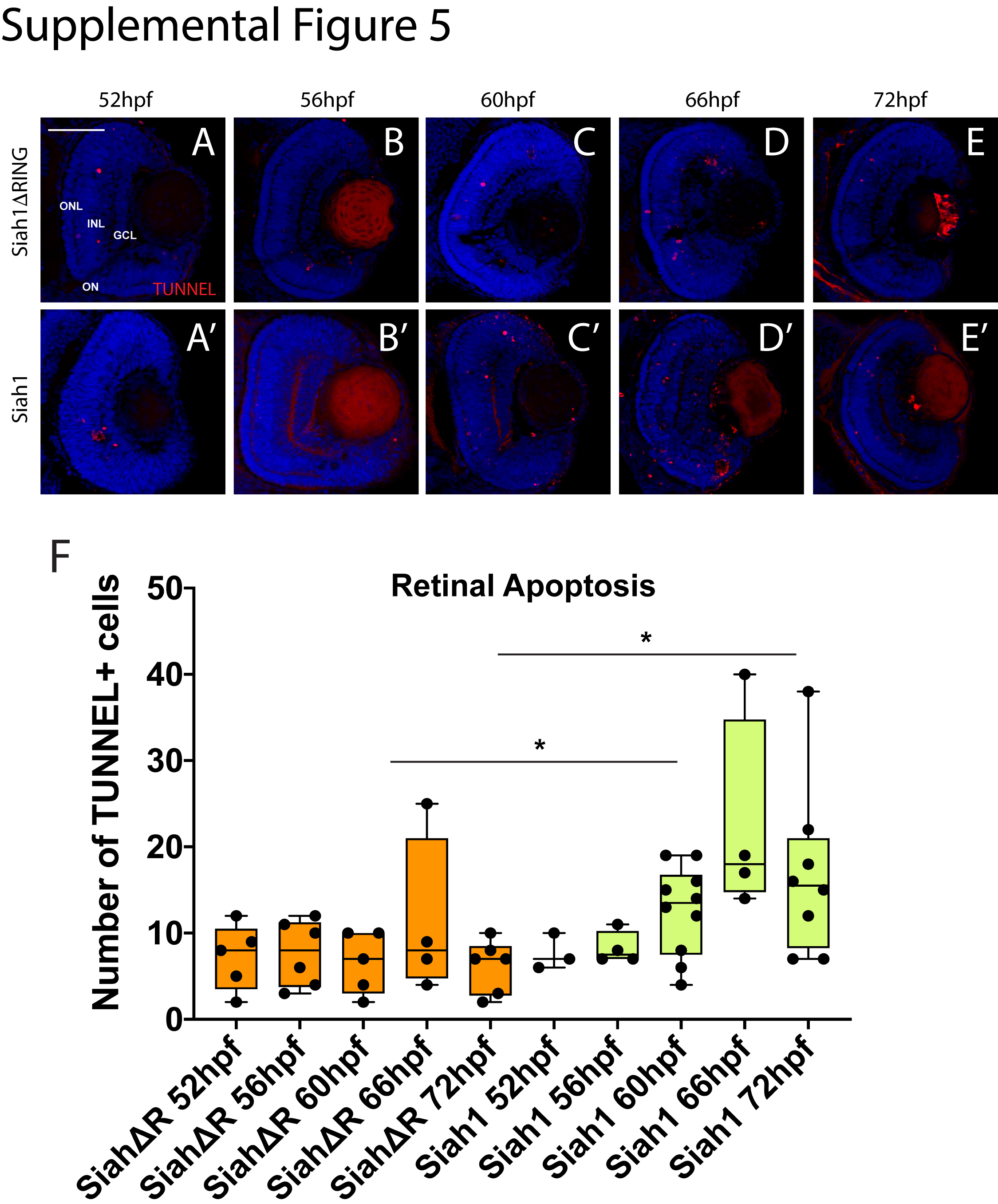

Supplement: Supplementary Figure 5 — Time course of retinal apoptosis following siah1 or siah1ΔRING heat shock. Retinal cryosections from heat shocked Tg[hsp70: siah1] (siah1), and Tg[hsp70:siah1ΔRING] (siah1ΔRING) embryos were analyzed using IHC for apoptosis using TUNEL staining (red) at various time points (A–E’). DNA was visualized using DAPI (blue). Heat shock induction of siah1ΔRING expression had little effect on induction of retinal apoptosis between 52–72 hpf (A–F). Heat shock induction of siah1 resulted in a significant increase of apoptotic cells in the retina beginning at 60 hpf and up to 72 hpf compared to siah1ΔRING (A’–E’, F). Each point on the graph represents counts from individual embryos (F). [file Image_5.TIF]

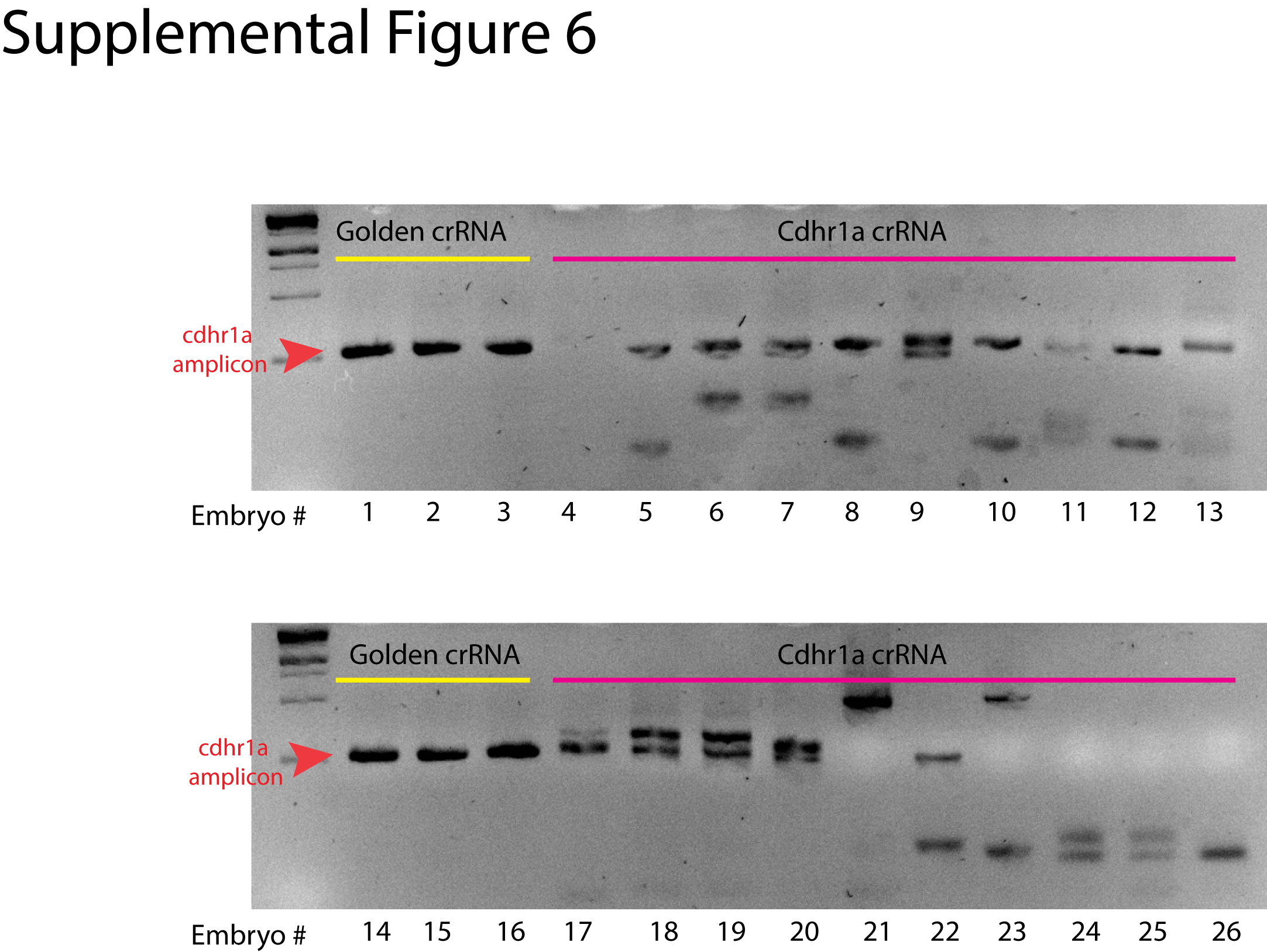

Supplement: Supplementary Figure 6 — Genomic PCR screen for CRISPR induced insertion or deletion. Genomic DNA collected from injected embryos was used to amplify ∼250 bp amplicon from cdhr1a coding sequence. Golden crRNA control injected embryos all display a strong 250 bp band (embryos 1–3, 14–16). When examining PCR products from cdhr1a crRNA injected embryos various combinations of insertion or deletion products were amplified (embryos 4–13 and 17–26. [file Image_6.TIF]
